# Supplementary material for: Coupling thermotolerance and high production of recombinant protein by CYR1N1546K mutation via cAMP signaling cascades
Source: Commun Biol. 2024 May 24;7:627. doi: 10.1038/s42003-024-06341-z (PMC11126729; doi:10.1038/s42003-024-06341-z)
Supplement: Supplementary file 2 — Supplementary Information [file 42003_2024_6341_MOESM2_ESM.pdf]

All the uncropped and unedited blot/gel images for each figure in the article file are provided below.

Supplementary Figure 1: The CYR1N1546K mutation improves recombinant protein yield in cells. These uncropped blot images are presented for Figure 3 in the article.

a Soybean hemoglobin LBA.

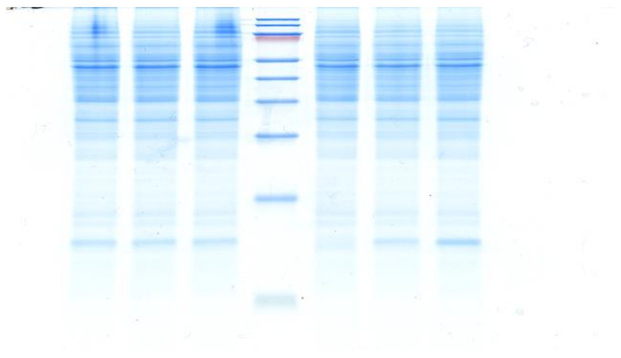

b Fowlpox virus coat protein HVP2. **c** Porcine circovirus coat protein PCV2.

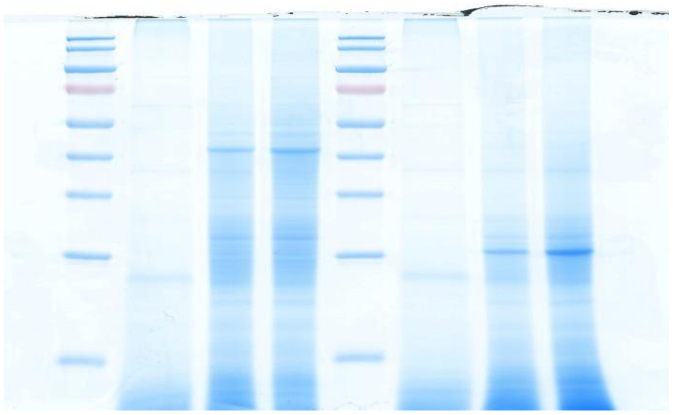

d AnFaeA esterase. **e**. Est1E esterase

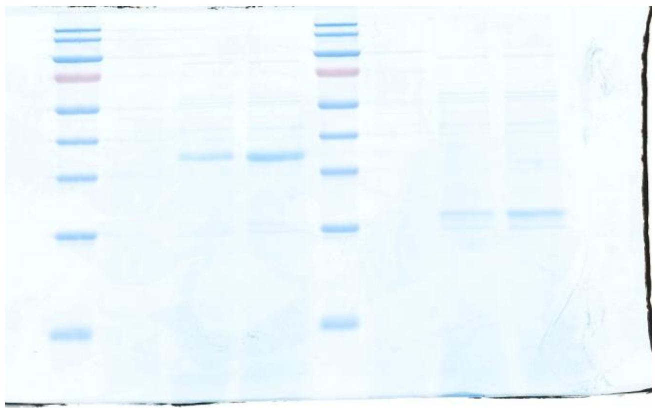

f Badgla glycosidase.

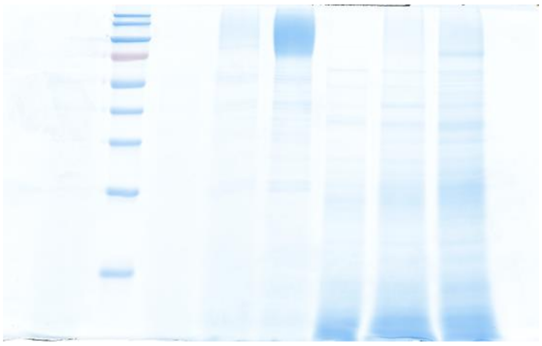

g The essential effect of CYR1N1546K mutation on recombinant LBA protein expression.

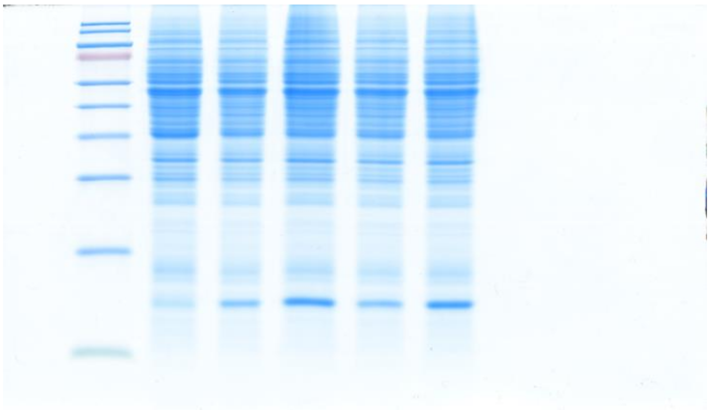

Supplementary Figure 2: Absolute quantification of protein. The absolute quantification of band intensity was estimated using GenoSens software by gray scanning analysis, employing varying concentrations of β-lactoglobulin as reference standards. The linear relationship established between β-lactoglobulin concentration and grayscale values facilitates the conversion of grayscale values into LBA concentrations.

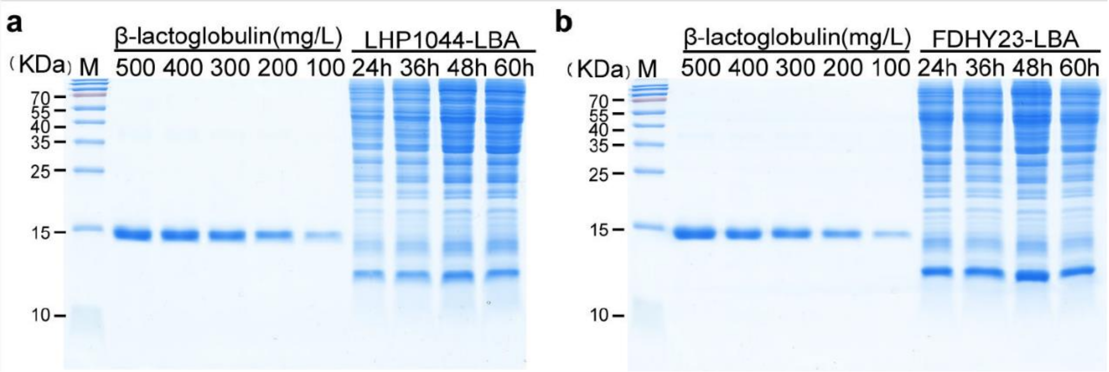

a For strain LHP1044 with recombinant LBA production. **b** For strain FDHY23 with recombinant LBA production,

Supplementary Figure 3: Comparison of recombinant LBA protein temporal production between FDHY23 and LHP1044 under shake flask cultivation and industrial fermentation conditions. These uncropped blot images are presented for Figure 4 in the article.

a Expressions of recombinant LBA protein under shake flask cultivation at 30°C.

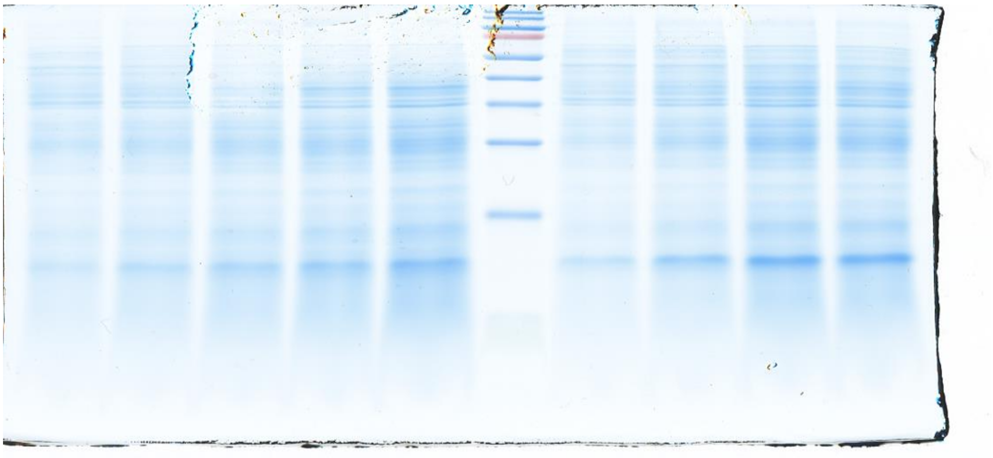

b Recombinant LBA protein yield during high-density fermentation with inorganic salts.

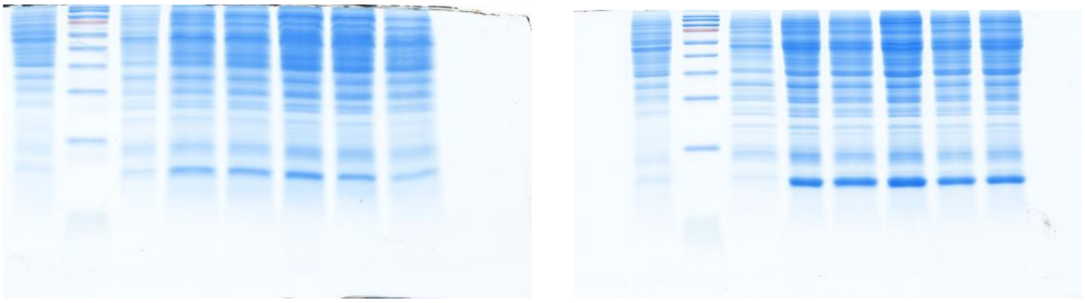

Supplementary Figure 4: Comparison of recombinant LBA protein production between FDHY23 and LHP1044 under high-temperature condition. These uncropped blot images are presented for Figure 5 in the article.

a Expressions of recombinant LBA protein at 46°C.

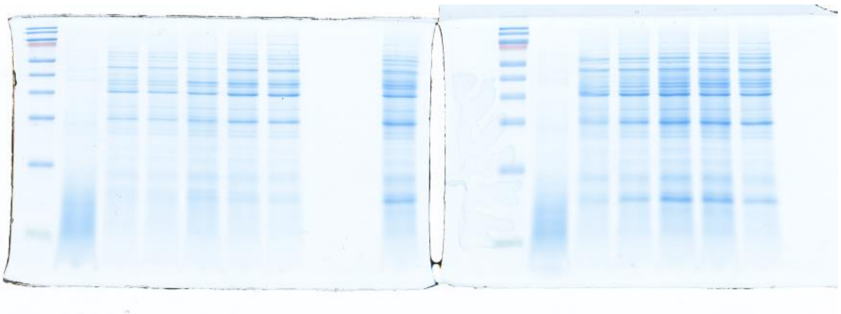

c Recombinant protein yield elevation at 46°C for the effect of CYR1N1546K mutation.

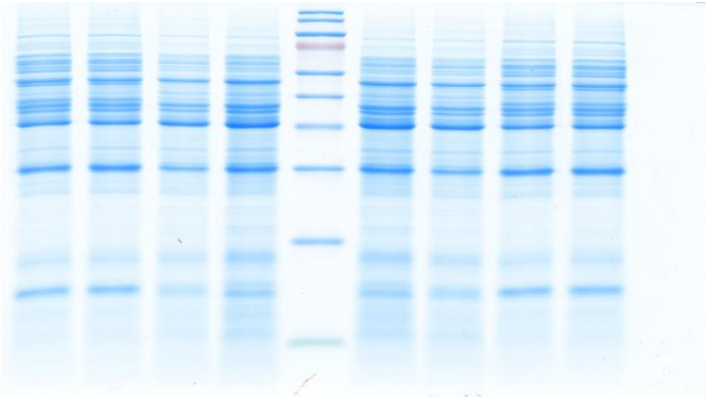

Supplementary Table 1. Amino acid sequences of the recombinant proteins produced in this study.

Protein name	Protein seq
LBA(NP_001235928.1)	MVAFTEKQDALVSSSFEAFKANIPQYSVVFYTSILEKAPAAKDLFSFL ANGVDPTNPKLTGHAEKLFALVRDSAGQLKASGTVVADAALGSVH AQKAVTDPQFVVVKEALLKTIKAAVGDKWSDELSRAWEVAYDELA AAIKKA
HVP2(OK167034.1)	MTNLQDQTQQIVPFIRSLLMPTTGPASIPDDTLEKHTLRSETSTYNL TVGDTGSGLIVFFPGFPGSIVGAHYILQSDGSYKFDQMLLTAQNLPA SYNYCRLVSRSLTVRSSTLPGGVYALNGTINAVTFQGSLSELTDVSY NGLMSATANINDKIGNVLVGEGVTVLSLPTSYDLGYVRLGDPIPAV GLDPKMVATCDSSDRPRVYTITAADNYQFSSQYKTGGVTITLFSANI DAITSLSVGGELVFKTSIQNLVLGATIYLIGFDGTAVITRAVAANNGL TAGIDNLMPFNLVIPTSEITQPITSIKLEIVTSKSDGQAGEQMSWSAS GSLAVTIHGGNYPGALRPVTLVAYERVAKGSVVTVAGVSNFELIPNP ELAKNLVTEYGRFDPGAMNYTKLILSERDRLGIKTVWPTREYTDFREY FMEVADLNSPLKIAGAFGFKDIIRALR
PCV2(ABV21950.1)	MTYPRRRFRRRRHRPRSHLGQILRRRPWLVHPRHRYRWRRKNGIF NTRLSRTIGYTVKKTTVRTPSWNVDMMRFNINDFLPPGGGSNPLTV PFEYYRIRKVKVEFWPCSPITQGDRGVGSTAVILDDNFVTKANALTY DPYVNYSSRHTITQPFSYHSRYFTPKPVLDRTIDYFQPNNKRNQLW LRLQTTGNVDHVGLGTAFENSIYDQDYNIRITMYVQFREFNLKDPP LNPK
Est1E(AXK50449.1)	MYIDCDGIKLNAYLDMPKNNPEKCPLCIIIHGFTGHSEERHIVAVQE TLNEIGVATLRADMYGHGKSDGKFEDHTLFKWLTNILAVVDYAKKL DFVTDIYMAGHSQGGLSVMLAAAMERDIIKALIPLSPAAMIPEIART GELLGLKFDPENIPDELEAWDGRKLKGNYARVAQTIRVEDFVDKYQ KPVLIVHGDQDEAVPYEFSVKFSKQYKNCKLVTIPGDTHCYDHHLEL VTEAVKEFMLEQIAK
AnFaeA(O42807.1)	MKQFSAKYALILLATAGQALAASTQGISEDLYNRLVEMATISQAAY

	ADLCNIPSTIIKGEKIYNAQTDINGWILRDDTSKEIITVFRGTGSDTNL QLDTNYTLTPFDTLPQCNDCEVHGGYYIGWISVQDQVESLVKQQA SQYPDYALTVTGHSLGASMAALTAAQLSATYDNVRLYTFGEPRSG NQAFASYMNDAFQVSSPETTQYFRVTHSNDGIPNLPPADEGYAH GGVEYWSVDPYSAQNTFVCTGDEVQCCEAQGGQGVNDAHTTYF GMTSGACTW

Supplementary Table 2. *K. marxianus* strains and mutants used in this study.

Name	Genotypes or description	Sources
LHP1044	MATα, ura3Δ	China General Microbiological Culture Collection Center (CGMCC, No 10621)
FDHY23	MATα, ura3Δ; exhibit excellent tolerance at a high temperature and efficiently express recombinant protein	This study
LHP1044-CYR1^{N1546K}	MATα, ura3Δ, CYR1::CYR1^{N1546K}	This study
LHP1044-LAP2^{G352R}	MATα, ura3Δ, LAP2::LAP2^{G352R}	This study
FDHY23-CYR1	MATα, ura3Δ, CYR1^{N1546K}::CYR1	This study

Supplementary Table 3. Plasmids used in this study.

Name	Description	Backbone	Application	Sources
LHZ531	ARS1, Cas9, gRNA, URA3	/	CRIPSR vector	[1]
1925-3	gRNA-1925-PAM-F/1925-PAM-R	LHZ531	CRISPR plasmid for the mutation of CYR1	This study
4931	gRNA-4931-PAM-F/4931-PAM-R	LHZ531	CRISPR plasmid for the mutation of LAP2	This study
pUKDN115	pKS, pKD1, P_{INU1}, T_{INU1}, URA3	/	Recombinant protein expression vector	[2]
pUKDN115-FTH1	pKS, pKD1, P_{INU1}, FYH1, T_{INU1}, URA3	pUKDN115	Intracellular expression of Fth1	This study
pUKDN115-LBA	pKS, pKD1, P_{INU1}, LBA, T_{INU1}, URA3	pUKDN115	Intracellular expression of LBA	This study
pUKDN115-HVP2	pKS, pKD1, P_{INU1}, HVP2, T_{INU1}, URA3	pUKDN115	Intracellular expression of HVP2	This study

pUKDN115-PCV2	pKS, pKD1, P_{INU1}, PCV2, T_{INU1}, URA3	pUKDN115	Intracellular expression of PCV2	This study
pUKDN132	pKS, pKD1, P_{INU1}, SS_{INU1}, T_{INU1}, URA3	/	Recombinant protein expression vector	[1]
pUKDN132-Est1E	pKS, pKD1, P_{INU1}, SS_{INU1}, Est1E, T_{INU1}, URA3	pUKDN132	Secretory expression of Est1E	This study
pUKDN132-AnFaeA	pKS, pKD1, P_{INU1}, SS_{INU1}, AnFaeA, T_{INU1}, URA3	pUKDN132	Secretory expression of AnFanA	This study
pUKDN132-Badgla	pKS, pKD1, P_{INU1}, SS_{INU1}, Badgla, T_{INU1}, URA3	pUKDN132	Secretory expression of Badgla	This study

Supplementary Table 4. Primers for plasmids and PCR used in this study.

Name	Sequence (5'-3')	Description
1925-PAM-F	TCACCCACCTCCAACAGGAAACG	gRNA for CRISPR plasmid 1925-3
1925-PAM-R	AACCGTTTCCTGTTGGAGGTGGG	gRNA for CRISPR plasmid 1925-3
4931-PAM-F	TCAGACCACTTCTGGCTAAACGA	gRNA for CRISPR plasmid 4931
4931-PAM-R	AACTCGTTTAGCCAGAAGTGGTC	gRNA for CRISPR plasmid 4931
1925-CF	ATTCTGTTTACACGGCAAATATTGG	PCR for amplifying the donor sequence of CYR1
1925-InCDs-R	CCTTATCTAAAGGTGTATTTTGTTCAGC	PCR for amplifying the donor sequence of CYR1
4931-CF	TCCATGCTAGAAGTTTGTTCCCATG	PCR for amplifying the donor sequence of LAP2
4931-InCDs-R	GAATAGGACGATTTGGTTGGAGGAG	PCR for amplifying the donor sequence of LAP2

Supplementary Table 5. Primers for qRT-PCR used in this study.

Name	Sequence (5'-3')	Description
2186-F(rt-pcr)	TACTACAACGCCAACAATGTGAAAGC	PCR for amplifying ATP23 in realtime PCR
2186-R(rt-pcr)	GGATTCGTCGAACTTCATATCGTCTG	PCR for amplifying ATP23 in realtime PCR
1981-F(rt-pcr)	CTCCGGTCAAGCTGCTGGTTACTCC	PCR for amplifying CYC1 in realtime PCR
1981-R(rt-pcr)	TGTCCTTTTCCTTCTTCAAACCACC	PCR for amplifying CYC1 in realtime PCR
3666-F(rt-pcr)	TCCATTAAGTATCACTAAAGTACCGAGCAG	PCR for amplifying CBS2 in

		realtime PCR
3666-R(rt-pcr)	CTCCGTATTGAAAGAATGATGGTTTGAAG	PCR for amplifying CBS2 in realtime PCR
2584-F(rt-pcr)	TTTGGTAAGGATTTGATCCCTCCA	PCR for amplifying HSP26 in realtime PCR
2584-R(rt-pcr)	CTTGGTCTCCTCGGTTGTAATGTCTG	PCR for amplifying HSP26 in realtime PCR
1914-F(rt-pcr)	ATGCTAATGGCTGGACTCAGTGCTG	PCR for amplifying EMC6 in realtime PCR
1914-R(rt-pcr)	GCCATATCGTTTGGATTGGCGTACC	PCR for amplifying EMC6 in realtime PCR
4955-F(rt-pcr)	GCTTTGGTGGATTTGGTGAAGATTTG	PCR for amplifying DNAJA1 in realtime PCR
4955-R(rt-pcr)	ACTTGCTTGTTCAAAGCCAACTTGG	PCR for amplifying DNAJA1 in realtime PCR
3825-F(rt-pcr)	GTCAACAACAAGCGTAGAGCATTCA	PCR for amplifying INP1 in realtime PCR
3825-R(rt-pcr)	CTCCAATCATTCCCATCAACAAAAGT	PCR for amplifying INP1 in realtime PCR
729-F(rt-pcr)	TGCGCAGAAGGGTGTAATCAGTAAGC	PCR for amplifying MRPL17 in realtime PCR
729-R(rt-pcr)	GGTCTTGAAACATCCGAGTTGAGCG	PCR for amplifying MRPL17 in realtime PCR
138-F(rt-pcr)	CTAAGGCTTTGGCTAAGCAAAACATCA	PCR for amplifying RPP1A in realtime PCR
138-R(rt-pcr)	TGTCATCATCGGATTCTTCCTTGGC	PCR for amplifying RPP1A in realtime PCR
3989-F(rt-pcr)	TGGCCAGTGCTGTCCAACATATTCT	PCR for amplifying CLU1 in realtime PCR
3989-R(rt-pcr)	CCTTTTCTTCTGGGTCCTCCTTTGC	PCR for amplifying CLU1 in realtime PCR
307-F(rt-pcr)	CTTTCCAGTGTATAGCTCCGGTGTCC	PCR for amplifying IFM1 in realtime PCR
307-R(rt-pcr)	GCTTGATAGCTTCTAGTGTTTGCGGC	PCR for amplifying IFM1 in realtime PCR
1199-F(rt-pcr)	CTAATTCCAACGTCCAAGCCACCTC	PCR for amplifying COG4 in realtime PCR
1199-R(rt-pcr)	TGCGCAATCATCGGATAATTATGCT	PCR for amplifying COG4 in realtime PCR
48-F(rt-pcr)	TGAAACCAAACGCATCGCACCC	PCR for amplifying SEC62 in realtime PCR
48-R(rt-pcr)	TGCACGCTTCATGTAAGGAGGCC	PCR for amplifying SEC62 in realtime PCR

4134-F(rt-pcr)	CGTTGGCATATGTGCTTTCGAGTTC	PCR for amplifying SEC11 in realtime PCR
4134-R(rt-pcr)	CACCACAACATCTCCCACATTATTCACC	PCR for amplifying SEC11 in realtime PCR
4741-F(rt-pcr)	ATCGACCATTGCAGAAAGGTTGCAG	PCR for amplifying YFH7 in realtime PCR
4741-R(rt-pcr)	TCACGAGTCTTGGCATCTGCCTCTG	PCR for amplifying YFH7 in realtime PCR
1313-F(rt-pcr)	CCGAAAGATTGATTGGTGATGCTGC	PCR for amplifying SSA1 in realtime PCR
1313-R(rt-pcr)	GGATTTGTGGCTTACCAGAAACCTC	PCR for amplifying SSA1 in realtime PCR
5149-F(rt-pcr)	GAAGGAAACTGCCGAGGCTTATTTG	PCR for amplifying SSA3 in realtime PCR
5149-R(rt-pcr)	TAGGCAATGGCAGCAGCAGTAGGTT	PCR for amplifying SSA3 in realtime PCR
3833-F(rt-pcr)	GCTGTTGGCACCAAGACCAGAAGAG	PCR for amplifying FBP1 in realtime PCR
3833-R(rt-pcr)	TGAACTTGAACGCAAACTGCAACGA	PCR for amplifying FBP1 in realtime PCR
1029-F(rt-pcr)	TGAGAAGAATGGAAATGGCTTGTGATG	PCR for amplifying PDA1 in realtime PCR
1029-R(rt-pcr)	CACCTCTCATGTAGGTGAAACCGTGAC	PCR for amplifying PDA1 in realtime PCR
2221-F(rt-pcr)	TTGGTCGTGGATCTTCTGAACTGGG	PCR for amplifying FMN1 in realtime PCR
2221-R(rt-pcr)	TTGTAGTTGACTTGCCTTCCATCCG	PCR for amplifying FMN1 in realtime PCR
2224-F(rt-pcr)	TGCAGGCAGACGTTCAAAGTTGGC	PCR for amplifying HEM3 in realtime PCR
2224-R(rt-pcr)	TGTCCACACCGCCTTACCACCAAAC	PCR for amplifying HEM3 in realtime PCR
1925-F(rt-pcr)	ATCAGTCGAAGGGCAACCTGAAACC	PCR for amplifying CYR1 in realtime PCR
1925-R(rt-pcr)	CGGTAGTGGAATGTCGCCGTCTG	PCR for amplifying CYR1 in realtime PCR
1385-F(rt-pcr)	CTATTTCACAAGGAGGCAGCAAGGC	PCR for amplifying BCY1 in realtime PCR
1385-R(rt-pcr)	GTTACGTCCACCAGCTAACCCAACG	PCR for amplifying BCY1 in realtime PCR
2756-F(rt-pcr)	GGAAGGGTGTATGTGGAACAGCAGC	PCR for amplifying YKL069W in realtime PCR
2756-R(rt-pcr)	ATGACGCCTACCGTCTCATTATCTTTAAC	PCR for amplifying YKL069W in realtime PCR

3269-F(rt-pcr)	TCACAGGACTTTGATTCGCCGTTTG	PCR for amplifying YKNTE1 in realtime PCR
3269-R(rt-pcr)	TTGATGATGGTGGTTGCACTGGACT	PCR for amplifying YKNTE1 in realtime PCR
YY605F	ATGGCCGCAAAGAAAACCCTTCAAG	PCR for amplifying SWC4 in realtime PCR
YY605R	ATTTGCTGCCTCTGCTGTGCAAATTG	PCR for amplifying SWC4 in realtime PCR

References:

1. Shi, T. et al. Characterization and modulation of endoplasmic reticulum stress response target genes in Kluyveromyces marxianus to improve secretory expressions of heterologous proteins. Biotechnol. Biofuels. 14, 236 (2021).

2. Yang, D. et al. Investigation of Kluyveromyces marxianus as a novel host for large-scale production of porcine parvovirus virus-like particles. Microb. Cell. Fact. 20, 24 (2021).
