# Supplementary material for: Coupling thermotolerance and high production of recombinant protein by CYR1N1546K mutation via cAMP signaling cascades
Source: Commun Biol. 2024 May 24;7:627. doi: 10.1038/s42003-024-06341-z (PMC11126729; doi:10.1038/s42003-024-06341-z)
Supplement: Supplementary file 3 — Description of Additional Supplementary Files [file 42003_2024_6341_MOESM3_ESM.pdf]

Description of Additional Supplementary Files

File name: Supplement Data 1

Description: The genomic mutations derived from DNAseq.

File name: Supplement Data 2

Description: RNAseq results for FDHY23 vs. LHP1044 in high temperature condition.

File name: Supplement Data 3

Description: RNAseq results for FDHY23 vs. LHP1044 in LBA production condition.

File name: Supplement Data 4

Description: upregulated processes and genes in FDHY23.

File name: Supplementary Data 5

Description: The source data behind the graphs in the paper.
