# Supplementary material for: Coupling thermotolerance and high production of recombinant protein by CYR1N1546K mutation via cAMP signaling cascades
Source: Commun Biol. 2024 May 24;7:627. doi: 10.1038/s42003-024-06341-z (PMC11126729; doi:10.1038/s42003-024-06341-z)
Supplement: Supplementary file 9 — Reporting Summary [file 42003_2024_6341_MOESM9_ESM.pdf]

nature portfolio

Corresponding author(s): Wenjuan Mo

Last updated by author(s): Apr 16, 2024

Reporting Summary

Nature Portfolio wishes to improve the reproducibility of the work that we publish. This form provides structure for consistency and transparency in reporting. For further information on Nature Portfolio policies, see our Editorial Policies and the Editorial Policy Checklist.

Statistics

For all statistical analyses, confirm that the following items are present in the figure legend, table legend, main text, or Methods section.

n/a	Confirmed	
☐	☒	The exact sample size (*n*) for each experimental group/condition, given as a discrete number and unit of measurement
☐	☒	A statement on whether measurements were taken from distinct samples or whether the same sample was measured repeatedly
☐	☒	The statistical test(s) used AND whether they are one- or two-sided<br>*Only common tests should be described solely by name; describe more complex techniques in the Methods section.*
☐	☒	A description of all covariates tested
☐	☒	A description of any assumptions or corrections, such as tests of normality and adjustment for multiple comparisons
☐	☒	A full description of the statistical parameters including central tendency (e.g. means) or other basic estimates (e.g. regression coefficient) AND variation (e.g. standard deviation) or associated estimates of uncertainty (e.g. confidence intervals)
☐	☒	For null hypothesis testing, the test statistic (e.g. *F*, *t*, *r*) with confidence intervals, effect sizes, degrees of freedom and *P* value noted<br>*Give P values as exact values whenever suitable.*
☒	☐	For Bayesian analysis, information on the choice of priors and Markov chain Monte Carlo settings
☐	☒	For hierarchical and complex designs, identification of the appropriate level for tests and full reporting of outcomes
☐	☒	Estimates of effect sizes (e.g. Cohen's *d*, Pearson's *r*), indicating how they were calculated

Our web collection on statistics for biologists contains articles on many of the points above.

Software and code

Policy information about availability of computer code

Data collection	Samples were sent to Sangon Biotech (Shanghai, China) for DNA sequencing, and to Biozeron (Shanghai, China) for RNA-seq analysis.
Data analysis	The reads were mapped to the reference genome of K.marxianus FIM1 (GenBank assembly accession: GCA_001854445.2). The SNPs or InDels in FDHY23 against LHP1044 were identified using the Genome Analysis Toolkit. The differential expression of genes between two samples was analyzed using edgeR (v3.8).

For manuscripts utilizing custom algorithms or software that are central to the research but not yet described in published literature, software must be made available to editors and reviewers. We strongly encourage code deposition in a community repository (e.g. GitHub). See the Nature Portfolio guidelines for submitting code & software for further information.

Data

Policy information about availability of data

All manuscripts must include a data availability statement. This statement should provide the following information, where applicable:

- Accession codes, unique identifiers, or web links for publicly available datasets
- A description of any restrictions on data availability
- For clinical datasets or third party data, please ensure that the statement adheres to our policy

The original DNA-seq and RNA-seq datasets presented in this study can be found in online NCBI Sequence Read Archive (SRA) repositories as follows: https://www.ncbi.nlm.nih.gov/bioproject/PRJNA1100266.

Research involving human participants, their data, or biological material

Policy information about studies with human participants or human data. See also policy information about sex, gender (identity/presentation), and sexual orientation and race, ethnicity and racism.

Reporting on sex and gender	None.
Reporting on race, ethnicity, or other socially relevant groupings	None.
Population characteristics	None.
Recruitment	None.
Ethics oversight	None.

Note that full information on the approval of the study protocol must also be provided in the manuscript.

Field-specific reporting

Please select the one below that is the best fit for your research. If you are not sure, read the appropriate sections before making your selection.

☒ Life sciences ☐ Behavioural & social sciences ☐ Ecological, evolutionary & environmental sciences

For a reference copy of the document with all sections, see nature.com/documents/nr-reporting-summary-flat.pdf

Life sciences study design

All studies must disclose on these points even when the disclosure is negative.

Sample size	To compare the high-temperature tolerance of LHP1044 and FDHY23, we conducted spotting and shaking tests. Each test was performed in biological triplicate. Samples for RNA-seq investigation were also performed in biological triplicate. Biological triplicate is commonly used in studies to minimize the impact of biological variability and increase the statistical power of the analysis. By repeating the experiment with three independent biological samples, it becomes possible to assess the consistency and generalizability of the results. This approach helps to account for individual differences and reduces the risk of drawing conclusions based on outliers or random variations. Additionally, using biological triplicates allows for the calculation of meaningful statistical measures, such as standard deviation and p-values, which provide a more robust and accurate assessment of the experimental findings. Overall, employing biological triplicate is a widely accepted practice in scientific research to ensure the validity and reliability of the results.
Data exclusions	No data were excluded from the analyses.
Replication	Spotting and shaking tests, as well as RNA-seq investigation, were conducted in biological triplicate to ensure the reliability and reproducibility of the results.
Randomization	Samples were allocated into the experimental group and the control group based on the sources of the strains.
Blinding	Blinding was not applicable in this study as the objective was to directly compare the properties of the mutated strain and the wild-type strain.

Reporting for specific materials, systems and methods

We require information from authors about some types of materials, experimental systems and methods used in many studies. Here, indicate whether each material, system or method listed is relevant to your study. If you are not sure if a list item applies to your research, read the appropriate section before selecting a response.

Materials & experimental systems

n/a	Involved in the study	
☒	☐	Antibodies
☐	☒	Eukaryotic cell lines
☒	☐	Palaeontology and archaeology
☒	☐	Animals and other organisms
☒	☐	Clinical data
☒	☐	Dual use research of concern
☒	☐	Plants

Methods

n/a	Involved in the study	
☒	☐	ChIP-seq
☒	☐	Flow cytometry
☒	☐	MRI-based neuroimaging

Eukaryotic cell lines

Policy information about cell lines and Sex and Gender in Research

Cell line source(s)	Kluyveromyces marxianus FIM1 strain used in this work was deposited at China General Microbiological Culture Collection Center (CGMCC, No 10621). The wild-type K. marxianus FIM1 strain LHP1044 and the mutant strain FDHY23 were used in this study, with FDHY23 being obtained through high-temperature selection.
Authentication	None of the cell lines used were authenticated.
Mycoplasma contamination	The cell lines were not tested for mycoplasma contamination.
Commonly misidentified lines (See ICLAC register)	No misidentified cell lines used in this study.

Plants

Seed stocks	None.
Novel plant genotypes	None.
Authentication	None.
